# Association between indoor residual spraying and the malaria burden in Zambia and factors associated with IRS refusals: a case-control study in Vubwi District

**DOI:** 10.1186/s13071-024-06328-z

**Published:** 2024-06-27

**Authors:** Wan-Xue Zhang, Yiguo Zhou, Elijah Tembo, Juan Du, Shan-Shan Zhang, Ting-Ting Wei, Ya-Qiong Liu, Chao Wang, Reuben Zulu, Busiku Hamainza, Fuqiang Cui, Qing-Bin Lu

**Affiliations:** 1 Department of Laboratorial Science and Technology & Vaccine Research Center, School of Public Health, Peking University, No. 38 Xue-Yuan Road, Haidian District, Beijing, China; 2 Department of Epidemiology and Biostatistics, School of Public Health, Peking University, Beijing, China; 3 Center for Infectious Diseases and Policy Research & Global Health and Infectious Diseases Group, Peking University, Beijing, China; 4 Department of Health Policy and Management, School of Public Health, Peking University, Beijing, China; 5 Ministry of Health, Vubwi District, Lusaka, Zambia; 6 National Malaria Elimination Centre, Lusaka, Zambia; 7 Key Laboratory of Epidemiology of Major Diseases (Peking University), Ministry of Education, Beijing, China

**Keywords:** Indoor residual spraying, Malaria, Retrospective study, Case-control study

## Abstract

**Background:**

Indoor residual spraying (IRS) has been implemented to prevent malaria in Zambia for several decades, but its effectiveness has not been evaluated long term and in Vubwi District yet. This study aimed to assess the association between IRS and the malaria burden in Zambia and Vubwi District and to explore the factors associated with refusing IRS.

**Methods:**

A retrospective study was used to analyze the association between IRS and malaria incidence in Zambia in 2001–2020 and in Vubwi District in 2014–2020 by Spearman correlation analysis. A case-control study was used to explore the factors associated with IRS refusals by households in Vubwi District in 2021. A logistic regression model was performed to identify factors associated with IRS refusals.

**Results:**

The malaria incidence reached its peak (391/1000) in 2001 and dropped to the lowest (154/1000) in 2019. The annual percentage change in 2001–2003, 2003–2008, 2008–2014, 2014–2018 and 2018–2020 was − 6.54%, − 13.24%, 5.04%, − 10.28% and 18.61%, respectively. A significantly negative correlation between the percentage of population protected by the IRS against the total population in Zambia (coverage) and the average malaria incidence in the whole population was observed in 2005–2020 (*r* = − 0.685, *P* = 0.003) and 2005–2019 (*r* = − 0.818, *P* < 0.001). Among 264 participants (59 in the refuser group and 205 in the acceptor group), participants with specific occupations (self-employed: OR 0.089, 95% CI 0.022–0.364; gold panning: OR 0.113, 95% CI 0.022–0.574; housewives: OR 0.129, 95% CI 0.026–0.628 and farmers: OR 0.135, 95% CI 0.030–0.608 compared to employees) and no malaria case among household members (OR 0.167; 95% CI 0.071–0.394) had a lower risk of refusing IRS implementation, while those with a secondary education level (OR 3.690, 95% CI 1.245–10.989) had a higher risk of refusing IRS implementation compared to those who had never been to school.

**Conclusions:**

Increasing coverage with IRS was associated with decreasing incidence of malaria in Zambia, though this was not observed in Vubwi District, possibly because of the special geographical location of Vubwi District. Interpersonal communication and targeted health education should be implemented at full scale to ensure household awareness and gain community trust.

**Supplementary Information:**

The online version contains supplementary material available at 10.1186/s13071-024-06328-z.

## Background

Malaria is an acute febrile disease caused by parasites transmitted by infected female *Anopheles* mosquitoes [1]. Malaria is characterized by symptoms such as fever, headache and chills, and it can develop into severe cases and even lead to death if not treated in time [2]. It is a major global public health problem with high morbidity and mortality globally [3, 4]. Though major achievements have been made in malaria control, the disease remains a significant cause of morbidity and mortality. Malaria is still endemic in around 90 countries with 247 million cases in 2021. The estimated number of malaria deaths has declined over time from 891,745 in 2001 followed by 651,325 in 2011 and 619,000 in 2021 [5, 6]. Sub-Saharan Africa accounted for about 90% of global malaria episodes and shoulders about 93% of all malaria deaths, and malaria was responsible for 30% to 50% of all outpatient visits to most health facilities and up to 50% of hospital admissions [7]. Malaria also has a crippling effect on the continent’s economic growth and perpetuates the cycles of poverty [8, 9].

In the past decades, significant efforts have been made to prevent malaria [10, 11]. One particularly effective approach has been the integrated vector management strategy, which encompasses a variety of interventions, such as indoor residual spraying (IRS) of all eligible structures, mass distribution of long-lasting insecticidal nets (LLINs) and insecticide-treated bed nets (ITNs) and intermittent presumptive treatment (IPT) of all antenatal mothers [12]. These measures have proven successful in controlling and eliminating malaria in many countries, with positive outcomes being observed across various regions [13, 14]. IRS is the application of a long-lasting, residual insecticide to a potential malaria vector resting on surfaces such as internal walls, eaves and ceilings of houses or structures (including domestic animal shelters) where such vectors might encounter the insecticides [15]. It has been used in sub-Saharan Africa for almost 80 years, since dichlorodiphenyltrichloroethane (DDT) was first introduced as an insecticide for IRS.

Several pilot projects in Africa demonstrated that malaria could be highly responsive to IRS with insecticides [10, 16]. By implementing comprehensive strategies to combat malaria, countries like South Africa, Swaziland and parts of Mozambique have made significant strides in reducing the mortality and morbidity from malaria [17]. Between 2000 and 2015, malaria infection rates halved while estimates suggested that malaria control interventions, especially ITNs and IRS, were estimated to have averted 663 million cases of malaria [10, 18]. Since 2000, 21 countries have eliminated malaria and 11 countries have been certified malaria-free by the World Health Organization (WHO).

Zambia is a landlocked country at the crossroads of Central, Southern and East Africa with an approximately 18.4 million population with a high malaria incidence despite the introduction and scaling up of malaria control interventions across the country. Zambia first initiated IRS with DDT in the 1950s but suspended the IRS program in the mid-1980s because of economic constraints and environmental concerns [19, 20]. After that, malaria prevention efforts in Zambia were relatively limited, with many activities focused on treating malaria [21]. In 2000, IRS was reintroduced with pyrethroids and DDT in two districts in Zambia with private funding [22]. In 2003, the Government of the Republic of Zambia began spraying and documenting to complement the private sector’s IRS campaigns in the southern and eastern provinces. Then, the Global Fund together with the US President’s Malaria Initiative (PMI) VectorLink Project provided support to the Ministry of Health (MOH) to scale up IRS. As evidence from insecticide resistance studies increases, those programs have adopted a rotation of insecticides [23]. In spray seasons 2014 to 2016, Zambia (Vubwi District included) used pirimiphos methyl with trade name Actelic 50 EC and then changed to Actellic 300 CS because of its longer residual effects of up to 9 months. From 2017 to 2019, Eastern, Lusaka, Western and Muchinga Provinces used SumiShield while Copperbelt Province used Fludora Fussion and Southern Province used DDT. In 2020 and 2021, all provinces used Fludora Fussion. These changes were due to recommendations from the technical working group based on insecticide resistance in the local vectors. IRS together with other anti-malaria methods is implemented to prevent malaria in Zambia and demonstrated protective effects [24, 25]. A 3-year evaluation of targeted IRS in northern Zambia showed that there was a moderate reduction in parasite prevalence in sprayed areas [26]. Analyses of three rounds of nationally representative cross-sectional surveys in Zambia indicated that the increases in interventions contributed to malaria reduction [27].

The WHO recommends a minimum coverage of 85% of all structures in areas targeted with IRS [28]. The National Malaria Elimination Centre of Zambia set a goal of achieving operational coverage of > 90% of eligible structures for their elimination strategy. Nonetheless, a previous study suggests that despite being used for decades,IRS coverage has not yet met WHO’s recommendation or the national strategy goal [29]. It is necessary to assess the relationship between the spraying of houses and malaria burden and to explore the factors of a low coverage of IRS in Zambia.

**Fig. 1:**
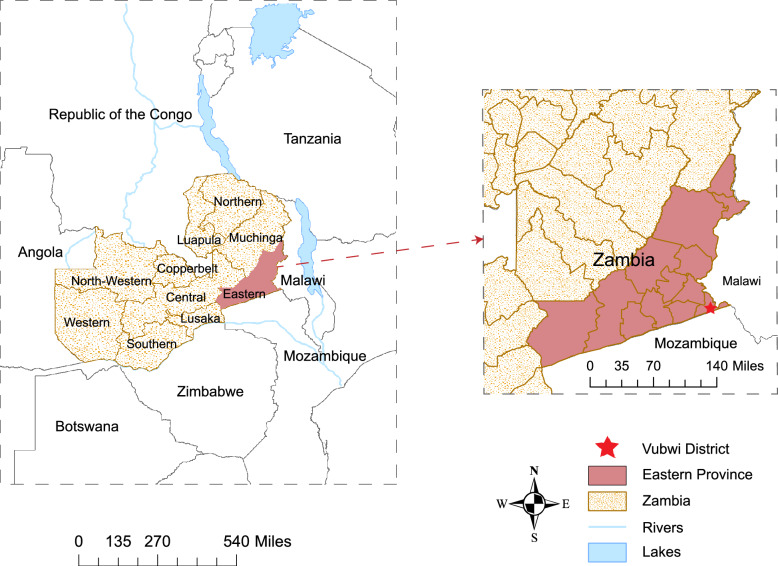
Location of Zambia and Vubwi District. The left shows the location of Zambia in Africa, with the Eastern Province highlighted. The red star on the right represents the location of Vubwi District in Eastern Province, which is on the border of Zambia, Malawi and Mozambique

Vubwi District is a rural district in the Eastern Province of Zambia with about 49,401 residents and 8243 households in 2021 (Fig. 1). The District Health Office divided the district into 12 catchment areas and put a rural clinic in every area to provide health services including IRS. IRS was initially conducted in Vubwi District in 2010–2012 when it was part of Chadiza District. Vubwi District became a separate district in 2012 and was targeted with IRS again beginning in 2014 after a gap of 2 years without it. The district was purposely selected in this study because of its disproportionately high malaria burden and low IRS coverage compared to other districts in the province (Health Management Information System, HMIS, 2019).

This study performed a retrospective study to evaluate the effectiveness of IRS on malaria incidence and deaths in Zambia and Vubwi District. Meanwhile, a case-control study was performed to explore the factors associated with refusing IRS in Vubwi District. These results might help the public health worker or government to refine the intervention and plan the resources in a professional manner.

## Methods

### Research design

A retrospective study and a case-control study were performed. The retrospective study was used to collect the annual malaria incidence, number of deaths and coverage of IRS in Zambia in 2001‒2020 and in Vubwi District in 2014–2020 to analyze the association between IRS and the malaria burden. The case-control study was used to investigate the factors associated with IRS refusals by households in Vubwi District in 2021.

### Study population

Households (families) in Vubwi District were recruited in the case-control study. The matching ratio was 1:4, and the refusers and acceptors were only matched by the same village. Refusers (cases) were selected from all the households that refused IRS in the most recent spraying campaign in the district. These refusers were extracted from the database at the District Health Office. The acceptors (controls) were the households that accepted IRS and were conveniently selected from the same villages as the refusers. The inclusion and exclusion criteria for participants were as follows: inclusion criteria: (i) willing to participate in the questionnaire survey; (ii) mentally sound and able to cooperate with the investigation; (iii) age ≥ 18 years. Exclusion criteria were: (i) cognitive impairment; (ii) incomplete questionnaire information.

### Data collection

Data used in the surveillance study were collected from the MOH at the Malaria Elimination Program. Further information and population data were collected from Zambian District Health Information System (DHIS) under VectorLink for the other aspects of coverage especially for the Eastern Province. Data on the use of ITNs in Zambia were collected from the Malaria Atlas Project (MAP) (https://malariaatlas.org/).

Data in the case-control study were collected mainly using a questionnaire survey. The questionnaire contained a series of questions including the demographic characteristics, IRS knowledge, acceptance of IRS and diagnosis of malaria during the past 6 months with blood tests carried out in the households. Data on IRS implementation and malaria incidence in Vubwi District were collected from the DHIS systems for VectorLink and Zambia HMIS, respectively.

### Sample size

The sample size of the case-control study was estimated, considering a 10% possibility of missing data. The proportion of no malaria cases in the past 6 months in the refuser group was assumed to be 0.1 under the null hypothesis and 0.3 under the alternative hypothesis. The proportion of no malaria cases in the past 6 months in the acceptor group was 0.1. The test statistic used was the two-sided Z-test with unpooled variance with a significance level of 0.05.

### Data analysis

Coverage of IRS in the whole population was calculated as the percentage of the number of the population protected by the IRS against the total population in Zambia. Coverage of IRS in the targeted population was calculated as the percentage of the number of the population protected by the IRS against the total population in the structures in that target community during the spraying exercise. The target population is determined by the number of people living in eligible structures to be sprayed. Eligibility criteria of a structure to be sprayed were as follows: people were living in the structure; there was a cluster of no less than 10 structures; the structure was reachable by a road; the people were willing to have their houses sprayed; the structure did not have metal walls.

All variables were reported using descriptive statistics, continuous variables were summarized as means and standard deviations (SD), and categorical variables were summarized as frequencies and proportions. To determine the difference between groups, an independent t-test, Chi-square test, Fisher’s exact test or nonparametric test was used where appropriate.

In the retrospective study, the joinpoint regression (JPR) model was used to define trends that were not constant over years. Statistically significant changes (joinpoint) in trends were estimated by using annual percentage change (APC). A positive value of APC indicated an increasing trend, while negative ones referred to a decreasing trend. Spearman correlation analysis was performed to explore the relationship between the annual coverage of IRS and malaria incidence.

In the case-control study, a logistic regression model was performed to identify variables that were associated with basic IRS knowledge, IRS implementation and malaria incidence. All data on demographic characteristics and basic knowledge scores were included in the univariate analysis. Biologically plausible variables with a *P* value < 0.10 in the univariate analysis were entered into a multivariate logistic regression model by a stepwise method.

All analyses were performed using R Programming Language V.3.2.2 (R Development Core Team, Vienna, Austria) and Stata 17 (Stata Corp. LP, College Station, TX, USA).

## Results

### Malaria incidence and IRS coverage in Zambia

**Fig. 2:**
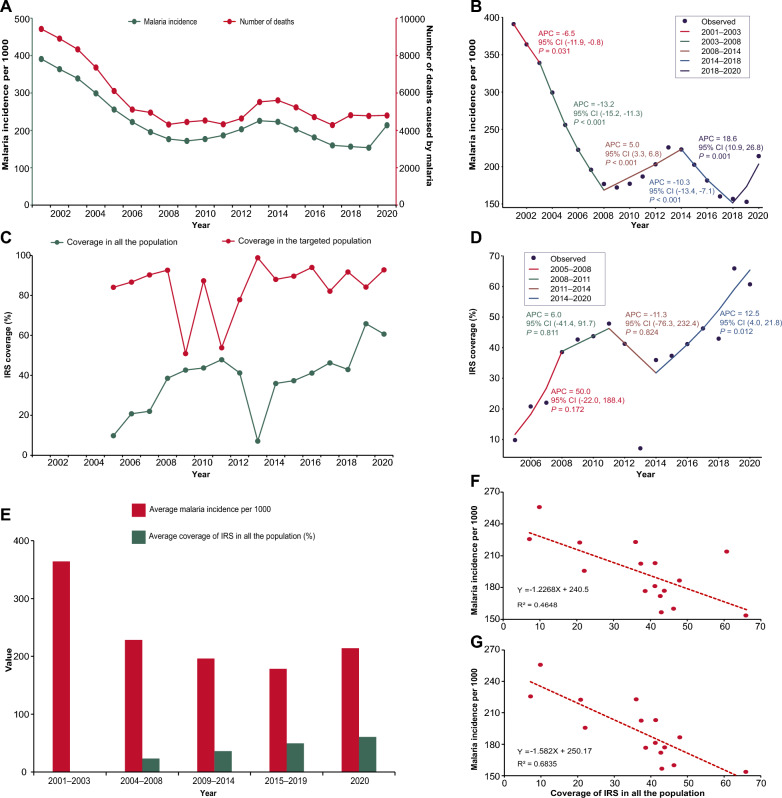
Relationship between IRS coverage and malaria burden in Zambia. **A** Malaria incidence and number of deaths in Zambia in 2001–2020. The green line presents the malaria incidence using the left y-axis. The red line presents the number of deaths using the right y-axis. **B** Joinpoint regression of malaria incidence for 2001–2020. A black point indicates the malaria incidence. The colored segments indicate the fitting values of the joinpoint regression. The text next to the segments shows the APC and its 95% CI of malaria incidence and the *P*-value at different stages. *APC* annual percentage change. *CI* confidence interval. **C** Coverage of IRS in the whole population and targeted population in Zambia during 2001–2020. The green line presents the coverage of IRS in the whole population. The red line presents the coverage of IRS in the targeted population. The IRS was reintroduced in Zambia from 2005. IRS, indoor residual spraying. **D** Joinpoint regression of coverage of IRS in the whole population during 2005–2020. A black point indicates the coverage of IRS in the whole population. The colored segments indicate the fitting values of the joinpoint regression. The text next to the segments shows the APC and its 95% CI of coverage of IRS in the whole population and the *P*-value at different stages. *IRS* indoor residual spraying, *APC* annual percentage change, *CI* confidence interval. **E** Average malaria incidence and average coverage of IRS in the whole population at different stages. The red column presents the average malaria incidence per 1000 at different stages. The green column presents the average coverage of IRS in the whole population at different stages. Since IRS has been implemented from 2005, there are no data in the period 2001–2003. **F** Correlation between the coverage of IRS in the whole population and malaria incidence in Zambia during 2005–2020. The red points present the coverage of IRS in the whole population and malaria incidence in Zambia from 2005 to 2020. The dotted red line is a trendline, and its formula and R^2^ are shown in the lower left corner. **G** Correlation between the coverage of IRS in the whole population and malaria incidence in Zambia during 2005–2019. The red points present the coverage of IRS in the whole population and malaria incidence in Zambia from 2005 to 2019. The dotted red line is a trendline, and its formula and R^2^ are shown in the lower left corner

The malaria incidence and number of deaths between 2001 and 2020 showed a similar trend (Fig. 2A). The malaria incidence peak occurred in 2001 with an incidence of 391 per 1000 and 9427 deaths, while the malaria incidence dropped to the lowest of 154 per 1000 in 2019. The fewest death cases were observed in 2017. During 2001–2009, the malaria incidence declined from 391 per 1000 to 172 per 1000, and the number of deaths from 9427 in 2001 to 4317 in 2008, respectively. In 2001–2003, a decrease in malaria incidence occurred (APC –6.54%), while during 2003–2008, a steeper decline in malaria incidence (APC − 13.24%) and the number of deaths occurred; at the same time, the government of Zambia scaled up IRS to all districts (Fig. 2B). The incidence of malaria and number of deaths began to fluctuate from 2009 to 2020. There was an increase (APC 5.04%) in malaria incidence during 2008–2014, followed by a decline (APC – 10.28%) during 2014–2018 and a rebound (APC 18.61%) during 2018–2020. The malaria incidence increased significantly in 2020 to 213 per 1000.

IRS was reintroduced and has been implemented in Zambia since 2000. The coverage of IRS varied in the targeted population and whole population during 2005–2020 (Fig. 2C). The coverage of IRS increased consistently from 84.10% in 2005 to 92.69% in 2008 among the targeted population and 9.82% in 2005 to 38.56% in 2008 among the whole population. Different trends occurred after 2008. For the targeted population, the coverage of IRS continued to fluctuate with the lowest rate of 50.92% in 2009 and the peak of 98.91% in 2013. For the whole population, the coverage of IRS continued to amplify with a slow increase (APC 6.0%) from 38.56% to 47.86% during 2008–2011 followed by a sharp decline (APC − 11.3%) until 2014 (Fig. 2D). The lowest rate was observed in 2013 with a coverage of 7.12%. Since 2014, there has been a stable increasing trend with a significant APC of 12.5%.

The Spearman correlation analysis showed a significantly negative correlation (*r* = − 0.685, *P* = 0.003) between the average coverage of IRS and the average malaria incidence in the whole population during 2005–2020 (Fig. 2E and F). A stronger correlation coefficient was observed (*r* = – 0.818, *P* < 0.001) from 2005 to 2019 when excluding the data from 2020 (Fig. 2G).

Use of ITNs in Zambia presented a fluctuating trend between 2010 and 2020 (Fig. S1A) and revealed an unsignificant correlation with malaria incidence (*r* = 0.074, Fig. S1B).

### Malaria incidence and coverage of IRS in Vubwi District

**Fig. 3:**
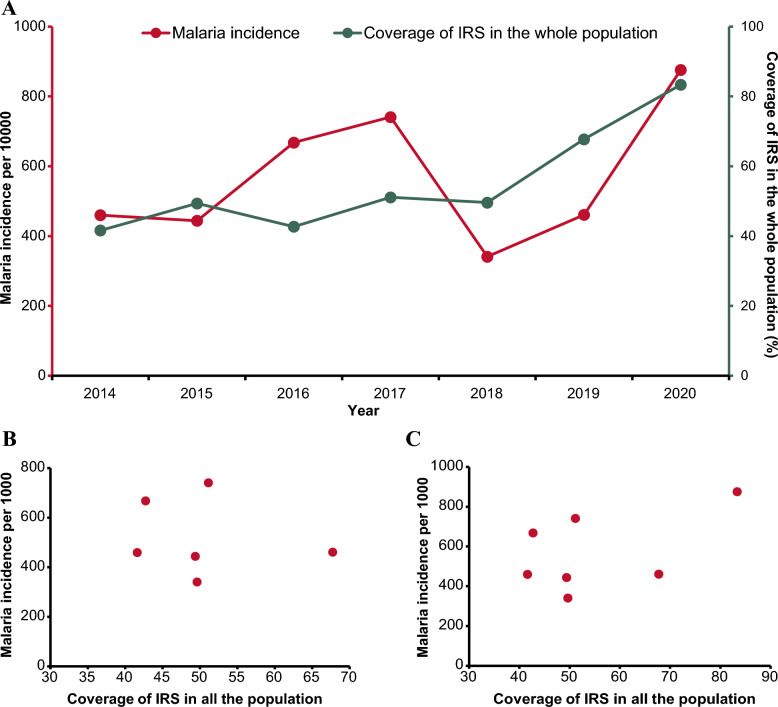
Relationship between IRS coverage and malaria burden in Vubwi District. **A** Malaria incidence and coverage of IRS in the whole population in Vubwi District during 2001–2020. The red line presents the malaria incidence using the left y-axis. The green line presents the coverage of IRS using the right y-axis. *IRS* indoor residual spraying. **B** Correlation between the coverage of IRS in the whole population and malaria incidence in Vubwi District during 2014–2019. The red points present the coverage of IRS in the whole population and malaria incidence in Vubwi District from 2014 to 2019. **C** Scatterplot of the coverage of IRS in the whole population and malaria incidence in Vubwi District during 2014–2020. The red points present the coverage of IRS in the whole population and malaria incidence in Vubwi District from 2014 to 2020

IRS coverage in Vubwi District kept an increasing trend during 2014–2020 from 41.60% to 83.35% (Fig. 3A) while the malaria incidence fluctuated considerably. During 2014–2017, with a slight decrease occurring in 2015, the malaria incidence kept amplifying until 2017. In 2018, there was a significant reduction in malaria incidence, with the lowest incidence observed at 341 per 1000. The malaria incidence in Vubwi District (876 per 1000) in 2020 was almost double that in 2019 (461 per 1000).

No significant correlations were detected between the IRS coverage and malaria incidence in Vubwi District in either the period of 2014–2019 or the period of 2014–2020 (Fig. 3B and C).

### Factors associated with IRS refusal

In the last spraying campaign, a total of 59 households spread across nine villages rejected IRS. During the investigation of the case-control study, some residents were not at home or refused to accept the investigation. The matching ratio for 31 refusers was adjusted from 1:4 to 1:3. Finally, 264 participants were recruited in this study, including 59 in the refuser group and 205 in the acceptor group. There were 81 (30.7%) participants aged between 18 and 25 years, 173 (65.5%) male, 120 (45.5%) married, 97 (36.7%) with a primary education level, 69 (26.1%) self-employed, 96 (36.4%) having 41–100 USD monthly income and 152 (51.7%) having children aged 5–14 years in their households (Table 1). Significant differences were observed in the participants’ age, gender, marital status, education level and occupation between the refuser and acceptor groups. On malaria intervention, 73 (27.7%) participants used LLINs while 191 (72.3%) participants used other interventions without a significant difference in the two groups (Table 2). A significant difference was observed between the two groups as there were more positive malaria blood tests in the refuser group.

**Table 1:** The basic characteristics of the participants

Characteristics	Total (*N* = 264)	Refuser group (*n* = 59)	Acceptor group (*n* = 205)	*P*
Age, years				0.023
18–25	81 (30.7)	19 (32.2)	62 (30.2)	
26–35	77 (29.2)	14 (23.7)	63 (30.7)	
36–45	40 (15.2)	12 (20.3)	28 (13.7)	
46–55	46 (17.4)	5 (8.5)	41 (20.0)	
≥ 56	20 (7.6)	9 (15.3)	11 (5.4)	
Marital status				< 0.001
Married	120 (45.5)	94 (45.9)	26 (44.1)	
Widowed	55 (20.8)	50 (24.4)	5 (8.4)	
Single	62 (23.5)	48 (23.4)	14 (23.7)	
Divorced	27 (10.2)	13 (6.3)	14 (23.7)	
Education				0.005
Primary	97 (36.7)	17 (28.8)	80 (39.0)	
Secondary	40 (15.2)	16 (27.1)	24 (11.7)	
Tertiary	46 (17.4)	14 (23.7)	32 (15.6)	
Never been to school	81 (30.7)	12 (20.3)	69 (33.7)	
Gender				0.026
Male	91 (34.5)	28 (47.5)	63 (30.7)	
Female	173 (65.5)	31 (52.5)	142 (69.3)	
Occupation				< 0.001
Employee	38 (14.4)	20 (33.9)	18 (8.8)	
Housewife	45 (17.1)	8 (13.6)	37 (18.0)	
Self-employed	69 (26.1)	10 (16.9)	59 (28.8)	
Farmer	48 (18.2)	11 (18.6)	37 (18.0)	
Gold panning	30 (11.4)	6 (10.2)	24 (11.7)	
Others	34 (12.9)	4 (6.8)	30 (14.6)	
Monthly income, USD				0.700
0–40	87 (33.0)	19 (32.2)	68 (33.2)	
41–100	96 (36.4)	20 (33.9)	76 (37.1)	
101–400	45 (17.1)	13 (22.0)	32 (15.6)	
≥ 401	36 (13.6)	7 (11.9)	29 (14.1)	
Age of children, years				0.154
0–5	142 (48.3)	39 (56.5)	103 (45.8)	
5–14	152 (51.7)	30 (43.5)	122 (54.2)	

*USD* United States dollar

**Table 2:** Use of other malaria interventions and malaria blood test results among households within the previous 6 months

Variable	Total, *N* (%)	Refuser group, *n* (%)	Acceptor group, *n* (%)	*P*
Other malaria interventions				
LLINs	73 (27.7)	17 (28.8)	56 (27.3)	0.951
Others	191 (72.3)	42 (71.2)	149 (72.7)	
Blood test				
Positive	154 (58.3)	46 (78.0)	108 (52.7)	0.001
Negative	110 (41.7)	13 (22.0)	97 (47.3)	

*LLINs* long-lasting insecticide bednets

The variables with a *P* value < 0.10 in the univariate analysis were put into the multivariate analysis to explore the factors associated with basic IRS knowledge, IRS implementation and malaria case diagnosis among participants (Tables 3, 4 and 5, Supplementary Material 1 Table S1-S3). The multivariate logistic regression model showed that being male (OR 2.892; 95% CI 1.307–6.399), of older age (36–45 years: OR 5.181, 95% CI 1.786–14.925; ≥ 56 years: OR 5.952, 95% CI 1.393–25.641) and farmers (OR 5.464; 95% CI 1.422–20.833) was associated with having less knowledge about IRS (Table 3). Participants with different occupations (self-employed: OR 0.089, 95% CI 0.022–0.364; gold panning: OR 0.113, 95% CI 0.022–0.574; housewives: OR 0.129, 95% CI 0.026–0.628; farmers: OR 0.135, 95% CI 0.030–0.608 compared to employees) and no malaria case among households (OR 0.167, 95% CI 0.071–0.394) were more likely to accept IRS implementation while a secondary education level (OR 3.690, 95% CI 1.245–10.989) was associated with having less acceptance of IRS implementation (Table 4). Participants aged 46–55 years (OR 4.180, 95% CI 1.310–13.337) were more likely to be diagnosed with malaria while those who had been sprayed in households (OR 0.210, 95% CI 0.096–0.458) had a lower risk of having been diagnosed with malaria (Table 5).

**Table 3:** Multivariable analysis of factors associated with basic IRS knowledge among participants

Variables	OR (95% CI)	*P*
Gender		
Male	Ref	
Female	0.346 (0.156–0.765)	0.009
Marriage		
Single	Ref	
Married	1.751 (0.608–5.051)	0.300
Divorced	1.248 (0.293–5.319)	0.764
Widowed	2.299 (0.556–9.524)	0.250
Age, years		
18–25	Ref	
26–35	1.949 (0.805–4.717)	0.139
36–45	5.181 (1.786–14.925)	0.002
46–55	1.621 (0.419–6.25)	0.484
≥ 56	5.952 (1.393–25.641)	0.016
Education		
Never been to school	Ref	
Primary	1.138 (0.519–2.494)	0.748
Secondary	0.808 (0.276–2.364)	0.697
Tertiary	1.167 (0.357–3.817)	0.799
Occupation		
Employee	Ref	
Housewife	2.132 (0.476–9.524)	0.323
Self-employed	2.653 (0.760–9.259)	0.126
Farmer	5.464 (1.422–20.833)	0.013
Gold panning	2.381 (0.551–10.309)	0.246
Others	0.865 (0.184–4.065)	0.854
Monthly income, USD		
0–40	Ref	
41–100	0.746 (0.337–1.653)	0.471
101–400	0.675 (0.228–2.000)	0.479
≥ 401	0.484 (0.142–1.650)	0.246
Had children aged < 5 years		
Yes	Ref	
No	0.504 (0.245–1.040)	0.064

*IRS* indoor residual spraying, *Ref* reference, *USD* US dollar, *OR* odds ratio, *CI* confidence interval

**Table 4:** Multivariable analysis of factors associated with IRS implementation among households

Variables	OR (95% CI)	*P*
Gender		
Male	Ref	
Female	0.589 (0.245–1.416)	0.237
Marriage		
Single	Ref	
Married	0.382 (0.128–1.144)	0.085
Divorced	2.062 (0.456–9.346)	0.347
Widowed	0.242 (0.041–1.406)	0.114
Age, years		
18–25	Ref	
26–35	0.442 (0.161–1.209)	0.112
36–45	0.985 (0.313–3.106)	0.980
46–55	0.256 (0.049–1.342)	0.107
≥ 56	2.825 (0.575–13.889)	0.201
Education		
Never been to school	Ref	
Primary	1.256 (0.476–3.322)	0.645
Secondary	3.690 (1.245–10.989)	0.018
Tertiary	1.033 (0.255–4.184)	0.963
Occupation		
Employee	Ref	
Housewife	0.129 (0.026–60.628)	0.011
Self-employed	0.089 (0.022–0.364)	0.001
Farmer	0.135 (0.030–0.608)	0.009
Gold panning	0.113 (0.022–0.574)	0.009
Others	0.124 (0.025–0.617)	0.011
Had children aged < 5 years		
Yes	Ref	
No	0.514 (0.223–1.186)	0.119
Had basic knowledge about IRS		
Yes	0.565 (0.243–1.312)	0.184
No	Ref	
Had malaria case among household in the past 6 months		
Yes	Ref	
No	0.167 (0.071–0.394)	< 0.001

*IRS* indoor residual spraying, *Ref* reference, *OR* odds ratio, *CI* confidence interval

**Table 5:** Multivariable analysis of factors associated with malaria case diagnosis among households

Variables	OR (95% CI)	*P*
Marriage		
Single	Ref	
Married	1.830 (0.858–3.903)	0.118
Divorced	0.920 (0.273–3.096)	0.893
Widowed	1.342 (0.450–4.002)	0.598
Age, years		
18–25	Ref	
26–35	1.599 (0.785–3.258)	0.196
36–45	1.221 (0.491–3.037)	0.668
46–55	4.180 (1.310–13.337)	0.016
≥ 56	0.850 (0.237–3.049)	0.802
Occupation		
Employee	Ref	
Housewife	1.559 (0.543–4.476)	0.409
Self-employed	0.918 (0.359–2.350)	0.859
Farmer	0.953 (0.352–2.577)	0.924
Gold panning	1.835 (0.587–5.740)	0.297
Others	1.025 (0.349–3.010)	0.965
Had children aged 5–14 years		
No/yes	0.811 (0.453–1.452)	0.481
Had been sprayed in households		
Yes/no	0.210 (0.096–0.458)	< 0.001

*Ref* reference, *OR* odds ratio, *CI* confidence interval

## Discussion

This is one of the few studies to elucidate the disease burden of malaria in recent years in Zambia and analyze its associations with IRS implementation. This study also selected a typical malaria high-epidemic district in Zambia to investigate the factors related to IRS refusals, in order to inform intervention measures to improve IRS coverage level among households. This study revealed an overall downward trend in malaria incidence and the number of deaths in Zambia during 2001–2020, with some slight increases observed. Additionally, an overall upward trend in the IRS coverage rate was found, albeit accompanied by fluctuations, in 2005–2020. The retrospective study demonstrated a negative correlation between the IRS coverage and malaria incidence in Zambia, though this correlation was not observed in Vubwi District. Sociodemographic characteristics of gender, age, education level and occupation were associated with IRS refusals.

Zambia is one of several countries that have switched from the goal of controlling malaria to achieving malaria elimination, which means changing from reducing the number of malaria cases to a very low level to reducing the number of indigenous cases to zero [30]. In the past 20 years, Zambia has made much progress in reducing the burden of malaria through a series of vector control interventions [21, 27, 31–33]. The negative correlation between IRS coverage and malaria incidence in Zambia from this study suggested that IRS was effective in controlling the spread of malaria. This finding was consistent with a research series conducted in Zambia and other countries concluding that the implementation of IRS would reduce parasite prevalence and in turn lower the malaria incidence [26]. Notably, there was a large drop in IRS coverage rate in the whole population in Zambia in 2013, which may be related to the reduction in IRS campaigns supported by the US President’s Malaria Initiative [34]. From 2014–2019, the malaria incidence decreased slightly while IRS coverage was being promoted, suggesting that continuous implementation and increased IRS coverage rate were critical for the control and elimination of malaria [35]. To date, malaria remains a major public health issue with high morbidity and mortality in Zambia. According to WHO, IRS can only be effective if at least 85% of eligible structures are spayed in a locality [28]. Nevertheless, the coverage rate of IRS in Zambia is still much lower than in other African countries such as Congo with a coverage rate of > 80% in 2020 [6]. Thus, continuous efforts to improve the IRS coverage rate to at least 85% are essential for Zambia to achieve the national goal of malaria elimination by 2030.

Notably, the malaria incidence in 2020 rebounded, which might be related to the lack of testing kits, such as malaria rapid diagnostic test kits, and mass LLIN distribution was delayed because of the coronavirus disease 2019 (COVID-19) pandemic [36]. The insufficient supply of diagnostic kits might cause clinicians to diagnose malaria cases relying only on clinical manifestations, inevitably leading to an increased number of false-positive cases. This increase in malaria incidence is not peculiar to Zambia alone but has occurred commonly in other countries in southern Africa. The COVID-19 pandemic may affect the availability of malaria control interventions, which will pose challenges to countries with a comparatively lower healthcare system capacity in reducing malaria morbidity and mortality [37, 38].

The notable effectiveness of IRS on the control of malaria in Zambia was identified at the country level, while this correlation was not observed in Vubwi District. The incidence of malaria in Vubwi District is higher than the average incidence in Zambia, which might be related to the special location of Vubwi District. This district was located on the border of Malawi, Zambia and Mozambique. Mozambique and Malawi have a high epidemic level of malaria, accounting for 4% and 1.8% of malaria cases in 2020, respectively [6]. The inflow of foreigners might lead to an upward trend in the incidence of malaria in this district. Importation of malaria parasites to Vubwi District from a high transmission zone is a major obstacle in reducing the malaria prevalence in areas aiming for elimination [39]. Thus, cross-border collaboration with neighboring countries should be established and strengthened. In this kind of high-risk setting, it is imperative to promote IRS to prevent malaria transmission. Therefore, the government should be devoted to obtaining community acceptance for IRS implementation in their living environment [40].

Previous studies concluded that misconceptions related to the use of IRS and other control measures were the factors reported to be potentially associated with persistence of malaria [41, 42]. A high malaria prevalence in Vubwi District was potentially exacerbated by socio-economic factors and socio-cultural practices among communities. The study showed that some factors were associated with people’s basic IRS knowledge, IRS implementation and malaria case diagnosis among households. Factors observed to be associated with having less knowledge of IRS were being male, of older age and a farmer. This may be because farmers (who are mostly male) are usually engaged in outdoor activities and have less access to health education compared to females and individuals with other occupations. In addition, the elderly may have difficulty understanding information about IRS. To improve and enhance community acceptance of IRS, multiple heath education programs targeted toward people with different backgrounds and concerns to enhance the level of community knowledge about IRS are recommended [43]. Additionally, participants with occupations other than employees, and those with no malaria cases in their households, were more likely to accept IRS implementation, while a lower education level was associated with less acceptance of IRS implementation. Thus, more precise strategies and measures targeting people with little education should be developed to raise their awareness of the benefits of implementing IRS in their households. Participants aged 46–55 years and with a lack of basic knowledge about IRS were more likely to be diagnosed with malaria. This suggested that the level of awareness and acceptance of IRS will affect the effectiveness of malaria prevention.

For most residents, the most common source of information about malaria prevention is mass media (television and radio) and health education by health facilities [44]. Studies have shown that social and behavior change communication (SBCC) advances individual exposure to malaria messages, acceptance and preventive practices through health education provided to schools or communities, which facilities the control of malaria [45, 46]. Delivering customized messages and creating a supportive environment that encourages individuals and communities to adopt positive health behaviors is essential for malaria control [47]. Besides, capacity-building activities should be provided to healthcare system actors to improve their knowledge and skills regarding SBCC programs [48]. The MOH in Zambia should design and implement context-specific and locally targeted community education interventions to address the knowledge gaps impeding effective malaria control in Vubwi District.

The study has several limitations. First, the observed associations between the coverage of IRS and malaria incidence were based on an ecological study, which was not sufficient to reflect the causal correlation. The reduction of malaria burden is not just the effect of IRS but the result of a series of interventions including the use of ITNs, LLINs, prevention during pregnancy with LLINs and malaria case management. This study could only provide exploratory results on the potential association between IRS coverage and malaria incidence; more evidence is needed to confirm this association. Second, population data of Vubwi District (census in 2010) used in this study might be underestimated. Besides, the sample size of the case-control study was not large enough to fully interpret the relevant factors associated with IRS refusal, and the representativeness of Vubwi District is limited. Further research is needed to confirm these predictors.

## Conclusions

IRS implementation in Zambia has demonstrated an opposite trend to the malaria burden. A continuous and higher IRS coverage rate should be reached for a better effect on malaria elimination in Zambia. In terms of IRS implementation, some sociodemographic characteristics such as lower education level were associated with IRS refusals. It is imperative that clear and constructive communication between the government and community and targeted health education should be implemented at full scale to ensure household awareness of IRS and gain community trust.

### Supplementary Information

Supplementary Material 1. Questionnaire

Supplementary Material 2: Table S1. Univariate analysis of factors associated with basic IRS knowledge among participants. Table S2. Univariate analysis of factors associated with IRS implementation among households. Table S3. Univariate analysis of factors associated with malaria case diagnosis. Fig. S1. Use of ITNs in Zambia between 2010 and 2020 and correlation with malaria incidence. (A) Use of ITNs in Zambia between 2010 and 2020. (B) Correlation of ITN usage and malaria incidence.

## Data Availability

The original dataset presented in this study is included in the supplementary material; further inquiries can be directed to the corresponding authors.

## Abbreviations

IRS: Indoor residual spraying
APC: Annual performance change
OR: Odds ratio
CI: Confidence interval
COVID-19: Coronavirus disease 2019
DDT: Dichlorodiphenyltrichloroethane
LLINs: Long-lasting insecticidal nets
MOH: Ministry of Health
IPT: Integrated presumptive treatment
ITNs: Insecticide-treated bed nets
WHO: World Health Organization
DHIS: District Health Information System
IPT: Integrated presumptive treatment
PMI: US President’s Malaria Initiative
SD: Standard deviations
JPR: Joinpoint regression
r: Correlation coefficient
*P*: *P*-value
