# Supplementary material for: Association between indoor residual spraying and the malaria burden in Zambia and factors associated with IRS refusals: a case-control study in Vubwi District
Source: Parasit Vectors. 2024 Jun 27;17:274. doi: 10.1186/s13071-024-06328-z (PMC11210042; doi:10.1186/s13071-024-06328-z)
Supplement: Supplementary file 1 — Supplementary Material 1. Questionnaire [file 13071_2024_6328_MOESM1_ESM.docx]

**Questionnaire**

**Instructions for research assistants**

1. Always introduce yourself before beginning the interview.
2. Explain the purpose of the study and ask for permission to do the interview.
3. Make the respondents sign or thumbprint the consent form before you start the interview.
4. Interviewer to read out the questions exactly the way they appear in the text.
5. Interpretation/translation should have the exact meaning as the text.
6. Assure confidentiality of collected information
7. Do not force a respondent to participate if they are reluctant or unwilling
8. Do not write the names of the respondents on the questionnaire
9. Write the appropriate response in the space provided.
10. Tick (√) the appropriate answer.
11. Thank the interviewee after the interviews.

**VILLAGE CODE………………..RESPONDENT CODE…………………………**

**NAME OF INTERVIEWER: .....................................................................**

**Section A: Demographic characteristics**

1. Sex of respondent

| 1 | Male |  |
| --- | --- | --- |
| 2 | Female |  |

1. Marital status

| 1 | Single |  |
| --- | --- | --- |
| 2 | Married |  |
| 3 | Divorced |  |
| 4 | Windowed |  |

1. Age

| 1 | 18-25 |  |
| --- | --- | --- |
| 2 | 26-35 |  |
| 3 | 36-45 |  |
| 4 | 46-55 |  |
| 5 | 1. nd above |  |

1. What is your level of education?

| 1 | Never been to school |  |
| --- | --- | --- |
| 2 | Primary |  |
| 3 | Secondary |  |
| 4 | Tertiary |  |

1. What is your occupation

| z | Employee |  |
| --- | --- | --- |
| 2 | Housewife |  |
| 3 | Self-employed |  |
| 4 | Farmer |  |
| 5 | Gold Panning |  |
| 6 | Others |  |

1. Monthly Income

| 1 | 0-40 USD |  |
| --- | --- | --- |
| 2 | 41-100 USD |  |
| 3 | 101-400 USD |  |
| 4 | 401 and above |  |

1. Children aged 0-5 years

| 1 | Yes |  |
| --- | --- | --- |
| 2 | No |  |

If yes how many? _____________

1. Children aged 5-14 years

| 1 | Yes |  |
| --- | --- | --- |
| 2 | No |  |

If yes how many? _____________

**Section B: IRS Knowledge**

1. Basic Knowledge about IRS? (1 if basic knowledge is known and 2 if not)

| 1 | Yes |  |
| --- | --- | --- |
| 2 | No |  |

**Section C: Acceptance of IRS**

1. Did spray operators visit your house and offer to spray it?

| 1 | Yes |  |
| --- | --- | --- |
| 2 | No |  |

1. If yes to question 10, was your house sprayed?

| 1 | Yes |  |
| --- | --- | --- |
| 2 | No |  |

1. If your house was sprayed, was spraying beneficial?

| 1 | Yes |  |
| --- | --- | --- |
| 2 | No |  |

1. If yes to question 11, what were the benefits of IRS?

| 1 | Reduction of mosquitoes density |  |
| --- | --- | --- |
| 2 | Reduction of Malaria episodes in the household |  |
| 3 | Others ( Specify) |  |

1. Apart from IRS, what other Malaria intervention do you used?

| 1 | LLINs |  |
| --- | --- | --- |
| 2 | Others ( Specify) |  |

1. If no to question 11, what were the reasons for refusing to have the house sprayed?

| 1 | Discoloring of inner house walls by insecticide |  |
| --- | --- | --- |
| 2 | The chemical smells bad |  |
| 3 | Difficulty in furniture’s movement |  |
| 4 | Bad behavior of spray operators |  |
| 5 | Others ( Specify) |  |

**Section D: Malaria Incidence**

1. Has anyone in your household had a blood test diagnosed malaria during the past six months?

| 1 | Yes |  |
| --- | --- | --- |
| 2 | No |  |

**Thank you for participating!**
