# Supplementary material for: Association between indoor residual spraying and the malaria burden in Zambia and factors associated with IRS refusals: a case-control study in Vubwi District
Source: Parasit Vectors. 2024 Jun 27;17:274. doi: 10.1186/s13071-024-06328-z (PMC11210042; doi:10.1186/s13071-024-06328-z)
Supplement: Supplementary file 2 — Supplementary Material 2: Table S1. Univariate analysis of factors associated with basic IRS knowledge among participants. Table S2. Univariate analysis of factors associated with IRS implementation among households. Table S3. Univariate analysis of factors associated with malaria case diagnosis. Fig. S1. Use of ITNs in Zambia between 2010 and 2020 and correlation with malaria incidence. (A) Use of ITNs in Zambia between 2010 and 2020. (B) Correlation of ITN usage and malaria incidence. [file 13071_2024_6328_MOESM2_ESM.docx]

**Supplementary Table 1. Univariate analysis on the factors impacting basic IRS knowledge among participants.**

| **Variables** | **OR (95% CI)** | ***P*** |
| --- | --- | --- |
| Gender |  |  |
| Male | Ref. |  |
| Female | 0.35 (0.201-0.610) | <0.001 |
| Marriage |  |  |
| Single | Ref. | Ref. |
| Married | 4.049 (1.767-9.259) | 0.001 |
| Divorced | 3.378 (1.133-10.101) | 0.029 |
| Widowed | 2.088 (0.793-5.495) | 0.136 |
| Age, years |  |  |
| 18-25 | Ref. |  |
| 26-35 | 2.037 (0.958-4.329) | 0.064 |
| 36-45 | 4.329 (1.855-10.101) | 0.001 |
| 46-55 | 1.007 (0.387-2.618) | 0.988 |
| ≥ 56 | 5.848 (2.041-16.667) | 0.001 |
| Education |  |  |
| Never been to school | Ref. |  |
| Primary | 0.947 (0.502-1.786) | 0.867 |
| Secondary | 0.705 (0.300-1.656) | 0.423 |
| Tertiary | 0.515 (0.217-1.222) | 0.132 |
| Occupation |  |  |
| Employee | Ref. |  |
| Housewife | 1.266 (0.430-3.731) | 0.669 |
| Self-employed | 1.563 (0.586-4.167) | 0.372 |
| Farmer | 5.236 (1.931-14.286) | 0.001 |
| Gold panning | 2.212 (0.724-6.757) | 0.163 |
| Others | 0.590 (0.157-2.227) | 0.437 |
| Monthly income, USD | |  |
| 0-40 | Ref. | . |
| 41-100 | 0.674 (0.363-1.250) | 0.21 |
| 101-400 | 0.409 (0.175-0.956) | 0.039 |
| ≥ 401 | 0.264 (0.093-0.746) | 0.012 |
| Had children aged < 5 years | |  |
| Yes | Ref. |  |
| No | 0.514 (0.295-0.895) | 0.019 |
| Had children aged 5-14 years | |  |
| Yes | Ref. |  |
| No | 1.479 (0.864-2.538) | 0.153 |

Note: IRS, indoor residual spraying. Ref, reference. USD, United States dollar. OR, odds ratio. CI, confidence interval.

**Supplementary Table 2. Univariate analysis of factors impacting IRS implementation among households.**

| **Variables** | **OR (95% CI)** | ***P*** |
| --- | --- | --- |
| Gender |  |  |
| Male | Ref. |  |
| Female | 0.491 (0.272-0.887) | 0.018 |
| Marriage |  |  |
| Single | Ref. |  |
| Married | 0.949 (0.454-1.98) | 0.888 |
| Divorced | 3.69 (1.412-9.615) | 0.008 |
| Widowed | 0.343 (0.115-1.025) | 0.055 |
| Age, years |  |  |
| 18-25 | Ref. |  |
| 26-35 | 0.725 (0.334-1.572) | 0.416 |
| 36-45 | 1.399 (0.598-3.268) | 0.439 |
| 46-55 | 0.398 (0.138-1.149) | 0.089 |
| ≥56 | 2.667 (0.962-7.407) | 0.059 |
| Education |  |  |
| Never been to school | Ref. |  |
| Primary | 1.222 (0.546-2.732) | 0.626 |
| Secondary | 3.831 (1.590-9.259) | 0.003 |
| Tertiary | 2.513 (1.046-6.061) | 0.039 |
| Occupation |  |  |
| Employee | Ref. |  |
| Housewife | 0.195 (0.072-0.526) | 0.001 |
| Self-employed | 0.153 (0.061-0.384) | <0.001 |
| Farmer | 0.268 (0.106-0.676) | 0.005 |
| Gold panning | 0.225 (0.075-0.675) | 0.008 |
| Others | 0.12 (0.035-0.407) | 0.001 |
| Monthly income, USD |  |  |
| 0-40 | Ref. |  |
| 41-100 | 0.942 (0.464-1.912) | 0.868 |
| 101-400 | 1.453 (0.640-3.300) | 0.372 |
| ≥ 401 | 0.864 (0.328-2.278) | 0.767 |
| Had children aged < 5 years |  |  |
| Yes | Ref. |  |
| No | 0.518 (0.283-0.948) | 0.033 |
| Had children aged 5-14 years | |  |
| Yes | Ref. |  |
| No | 1.420 (0.794-2.545) | 0.237 |
| Had basic knowledge about IRS |  |  |
| Yes | 1.545 (0.834-2.864) | 0.167 |
| No | Ref. |  |
| Used other interventions |  |  |
| LLINs | Ref. |  |
| Others | 0.929 (0.489-1.764) | 0.821 |
| Had malaria case among household in the past 6 months | | |
| Yes | Ref. |  |
| No | 0.315 (0.160-0.617) | 0.001 |

Note: IRS, indoor residual spraying. Ref, reference. USD, United States dollar. LLINs, Long-Lasting Insecticide bed-nets. OR, odds ratio. CI, confidence interval.

**Supplementary Table 3. Univariate analysis of factors impacting malaria case diagnosis.**

| **Variables** | **OR (95% CI)** | ***P*** |
| --- | --- | --- |
| Gender |  |  |
| Male | Ref. |  |
| Female | 1.518 (0.909-2.535) | 0.111 |
| Marriage |  |  |
| Single | Ref. |  |
| Married | 2.160 (1.158-4.030) | 0.015 |
| Divorced | 1.886 (0.753-4.718) | 0.175 |
| Widowed | 2.456 (1.161-5.194) | 0.019 |
| Age, years |  |  |
| 18-25 | Ref. |  |
| 26-35 | 1.598 (0.851-3.002) | 0.145 |
| 36-45 | 1.615 (0.749-3.483) | 0.221 |
| 46-55 | 3.427 (1.531-7.669) | 0.003 |
| ≥ 56 | 1.077 (0.405-2.866) | 0.882 |
| Education |  |  |
| Never been to school | Ref. |  |
| Primary | 0.730 (0.398-1.339) | 0.31 |
| Secondary | 0.616 (0.286-1.330) | 0.218 |
| Tertiary | 0.725 (0.346-1.518) | 0.394 |
| Occupation |  |  |
| Employee | Ref. |  |
| Housewife | 0.837 (0.334-2.092) | 0.703 |
| Self-employed | 0.448 (0.195-1.029) | 0.059 |
| Farmer | 0.502 (0.206-1.219) | 0.128 |
| Gold panning | 1.077 (0.381-3.041) | 0.889 |
| Others | 0.585 (0.223-1.530) | 0.274 |
| Monthly income, USD |  |  |
| 0-40 | Ref. |  |
| 41-100 | 0.865 (0.480-1.559) | 0.63 |
| 101-400 | 0.921 (0.444-1.912) | 0.825 |
| ≥ 401 | 1.058 (0.477-2.343) | 0.89 |
| Had children aged < 5 years |  |  |
| No/Yes | 0.820 (0.502-1.340) | 0.428 |
| Had children aged 5-14 years |  |  |
| No/Yes | 0.627 (0.382-1.029) | 0.065 |
| Had basic knowledge about IRS |  |  |
| No/Yes | 0.777 (0.449-1.346) | 0.369 |
| Had been sprayed in households |  |  |
| Yes/No | 0.315 (0.160-0.617) | 0.001 |
| Used other interventions |  |  |
| LLINs | Ref. |  |
| Others | 1.538 (0.893-2.649) | 0.12 |

Note: IRS, indoor residual spraying. Ref, reference. USD, United States dollar. LLINs, Long-Lasting Insecticide bed-nets. OR, odds ratio. CI, confidence interval.


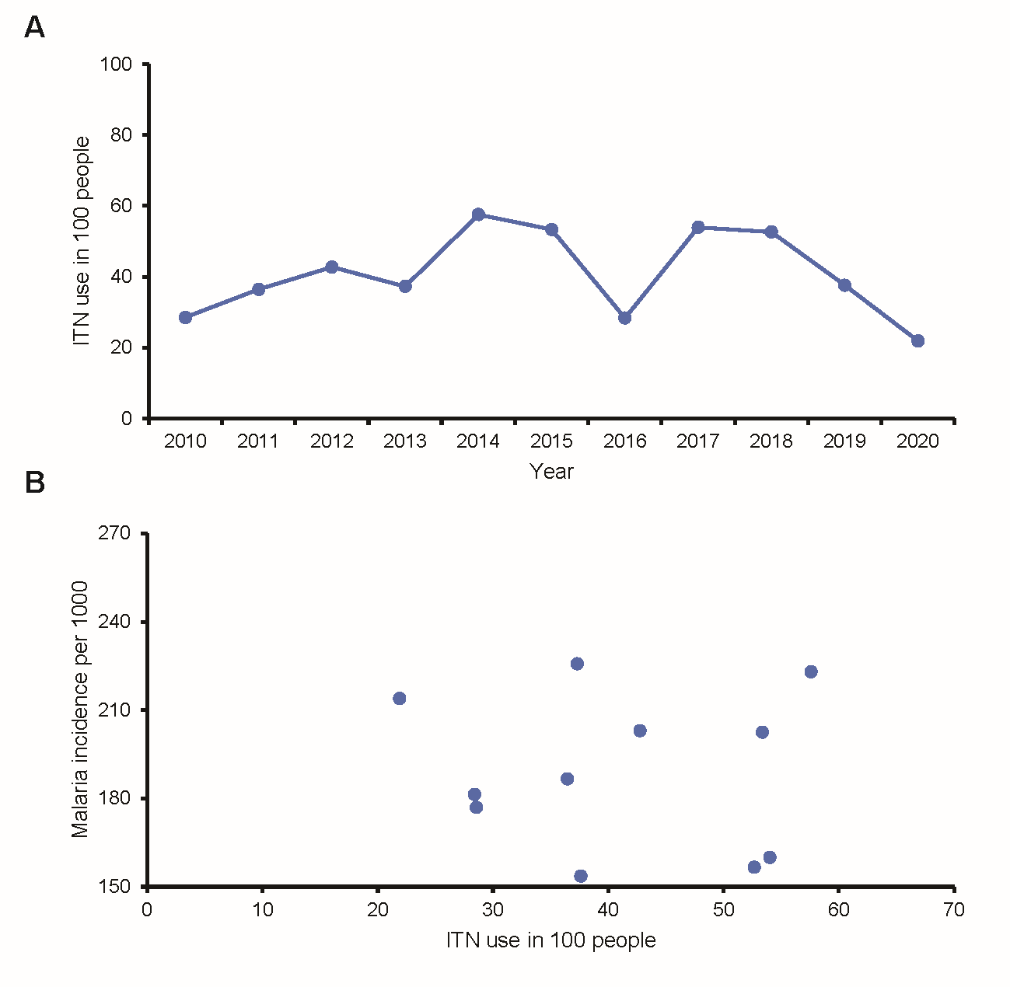


**Fig. S1.** The use of ITNs in Zambia between 2010 and 2020 and the correlation with malaria incidence. (A) The use of ITNs in Zambia between 2010 and 2020. (B) the correlation of ITN usage and malaria incidence.
